# Estradiol-sensitive projection neurons in the female rat preoptic area

**DOI:** 10.3389/fnins.2015.00067

**Published:** 2015-03-24

**Authors:** Yasuo Sakuma

**Affiliations:** Laboratory of Physiology, University of Tokyo Health SciencesTokyo, Japan

**Keywords:** sexual behavior, estradiol, preoptic, ventral tegmental area, central gray, lordosis reflex

## Abstract

Electrical stimulation of the preoptic area (POA) interrupts the lordosis reflex, a combined contraction of back muscles, in response to male mounts and the major receptive component of sexual behavior in female rat in estrus, without interfering with the proceptive component of this behavior or solicitation. Axon-sparing POA lesions with an excitotoxin, on the other hand, enhance lordosis and diminish proceptivity. The POA effect on the reflex is mediated by its estrogen-sensitive projection to the ventral tegmental area (VTA) as shown by the behavioral effect of VTA stimulation as well as by the demonstration of an increased threshold for antidromic activation of POA neurons from the VTA in ovariectomized females treated with estradiol benzoate (EB). EB administration increases the antidromic activation threshold in ovariectomized females and neonatally castrated males, but not in neonatally androgenized females; the EB effect is limited to those that show lordosis in the presence of EB. EB causes behavioral disinhibition of lordosis through an inhibition of POA neurons with axons to the VTA, which eventually innervate medullospinal neurons innervating spinal motoneurons of the back muscle. The EB-induced change in the threshold or the axonal excitability may be a result of EB-dependent induction of BK channels. Recordings from freely moving female rats engaging in sexual interactions revealed separate subpopulations of POA neurons for the receptive and proceptive behaviors. Those POA neurons engaging in the control of proceptivity are EB-sensitive and project to the midbrain locomotor region (MLR). EB thus enhances lordosis by reducing excitatory neural impulses from the POA to the VTA. An augmentation of the POA effect to the MLR may culminate in an increased locomotion that embodies behavioral estrus in the female rat.

## Overview

Protracted electrical stimulation of the ventrolateral part of the ventromedial nucleus of the hypothalamus (VMN) at low frequencies has been found to cause lasting facilitation of the lordosis reflex in female rats in the estrus, a combined contraction of the longissimus and other back muscles caused by touch-pressure stimulation on the flank-perineal skin given by male partners (Pfaff and Sakuma, 1979). The resultant dorsiflexion of the female trunk allows penile penetration. A recent study replicated the effectiveness of low frequency stimulation, albeit by optogenic stimulation in male mice, to elicit sexual behavior or aggression from the ventrolateral VMN at different thresholds (Lin et al., 2011). The effects of electrical stimulation at low frequencies may be compatible with the scalable control of mounting and attack at different optogenic stimulation thresholds at the similar frequency range around 10 Hz (Lee et al., 2014).

## Systemic estrogen is needed for effective VMN stimulation

Systemic treatment with submaximal doses of estrogen, in particular estradiol benzoate (EB), was needed for electrical stimulation of the VMN to facilitate lordosis in the ovariectomized female rats. EB-induced increase in the excitability of VMN neurons does not fully explain the requirement of systemic EB to stimulation-bound facilitation of lordosis, because VMN stimulation does not promote lordosis in the absence of systemic EB, even at stronger currents. The VMN contains estrogen receptor (ER) α positive projection neurons to the midbrain, but ERα positive neurons are also present in the preoptic area (POA), medial amygdala, midbrain central gray (CG), and lateral septum, to name but a few (Simerly et al., 1990; Doncarlos et al., 1991). In the periphery, EB-induced enlargement of the cutaneous sensory field pertinent to the induction of lordosis has been shown (Kow and Pfaff, 1973).

The medial amygdala exerts an estrogen-dependent facilitatory effect on lordosis, evidence that is based on lesion of the structure (Rajendren and Moss, 1993) and resection of its efferents in the stria terminalis (Takeo et al., 1995). Significant reduction the lordosis quotient following lesion of the amygdala was, however, detected only in the response to repeated coital stimulation. Fos immunohistochemistry attributed the effect secondary to the diminished activation of gonadotropin-releasing hormone neurons. Thus, the medial amygdala cannot be a principal site for estrogen action on the lordosis reflex. The lateral septum is also an origin of a lordosis-inhibiting efferents (Yamanouchi and Arai, 1990), and EB implants in this structure releases the behavioral inhibition (Satou and Yamanouchi, 1999). Morphologically, however, only a small number of estrogen receptor immunoreactive cells have been visualized in this structure (Yokosuka et al., 1997).

## The POA as a target of estrogen action

Whereas, the VMN is known to play a key role in the lordosis reflex and other components of estrogen-dependent female sexual behavior, the POA has more often been associated with male behavior and is not traditionally been considered to be vital in the regulation of female behavior. Several earlier studies have shown, however, that the POA is primarily an inhibitory structure for the lordosis reflex. Stereotaxic implantation of minute amount of crystalline EB either in the VMN or the POA supplements a subthreshold EB given systemically to induce lordosis (Barfield and Chen, 1977). Although larger doses were needed to induce lordosis by implants in the POA than in the VMN, this observation has shown that the POA is a target site of estrogen action to induce lordosis.

Pharmacological disruption of aminergic neurotransmission in the POA has been found to promote lordosis (Ward et al., 1975; Carter et al., 1978). Intracerebral implantation of the anti-estrogens in the preoptic and anterior hypothalamic continuum has also been found to antagonize systemic EB, which results in a dramatic inhibition of lordosis (Luttge, 1976). Additionally, lesions in the dorsal POA have been found to produce a significant increase in lordosis (Nance et al., 1977). It is worth noting that the CG receives dense projection from the rostral and dorsal parts of the POA (Morrell et al., 1981; Swanson et al., 1987).

## POA stimulation and lordosis

**Figure 1:**
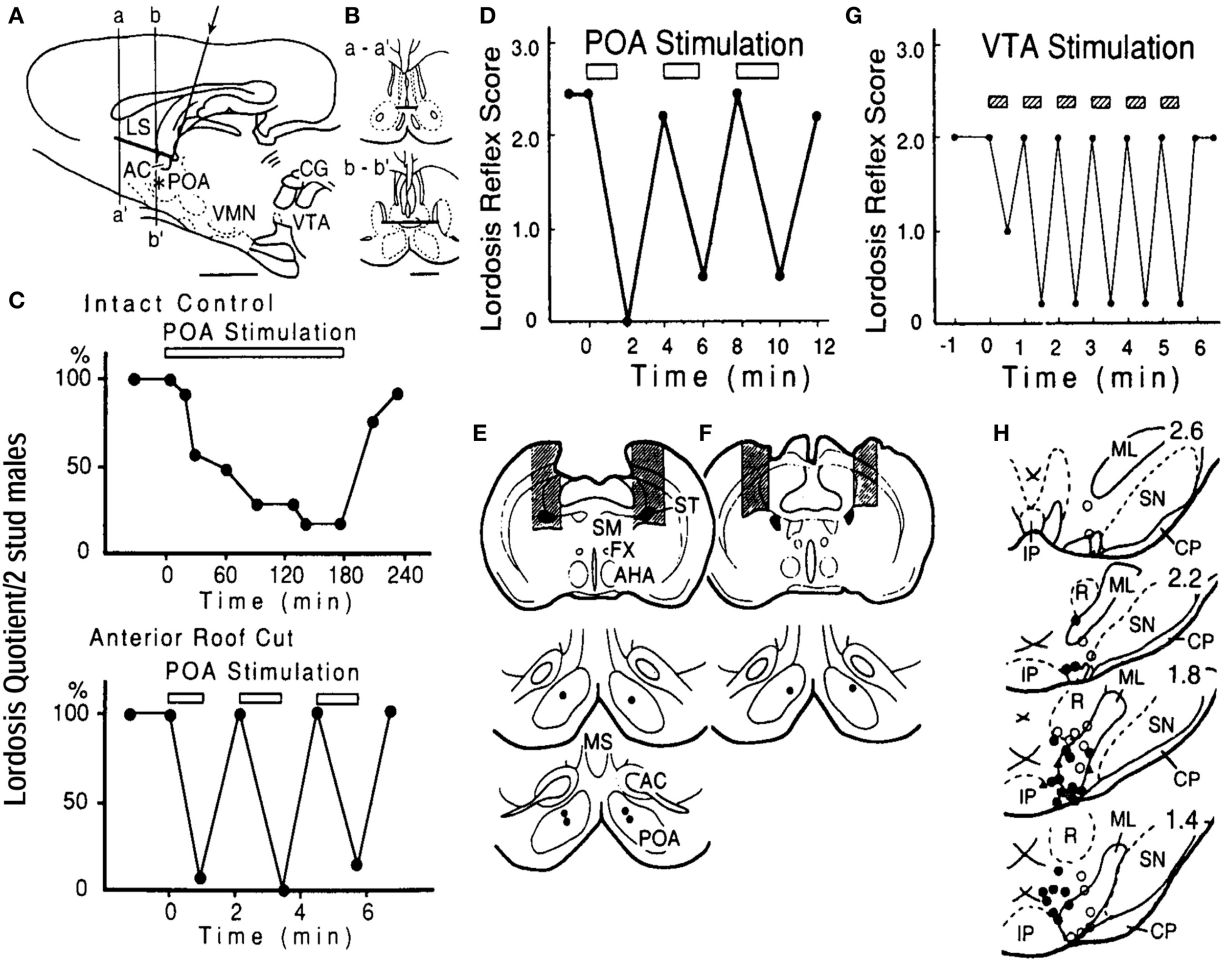
**(A)** Locations and the extent of the roof cut of the POA in the sagittal **(A)** and frontal **(B)** planes. An L-shaped wire was inserted in the midline (arrow) and rotated 180°. The asterisk shows the stimulation site in the POA. AC, anterior commissure; LS, lateral septum; other abbreviations are in the text. **(C)** Lordosis reflex suppression during bilateral POA stimulation in the intact control (top) and roof-cut (bottom) animals. POA was stimulated at 100 Hz for periods indicated by the bar in each panel. Stimulus intensity was 100 μA per electrode. Note different time scales. **(D)** Interruption of lordosis by POA stimulation in rats with bilateral cuts of the stria terminalis (ST, shaded areas in **E**, top panel). POA stimulation was ineffective when the ST was spared (shaded areas in **F**). Stimulation sites are in lower panels in **(E)** and **(F)** (Takeo et al., 1993). **(G)** Time course of suppression of lordosis by electrical stimulation of the VTA. Pulses of 50 μA, 100 Hz were applied in 30-s trains during the period indicated by shaded bars. **(F)** Locations of stimulation sites in the VTA and adjacent tegmentum plotted on sections 400 um apart. Filled circles, suppression exceeding 50% of prestimulation lordosis reflex score at current intensity below 50 μA; open circles, suppression under 50% or no effect. Abbreviations: CP, cerebral peduncle: IP, interpeduncular nucleus: ML, medial lemniscus: p, pons: R, red nucleus: SN, substantia nigra; III, oculomotor nerve (Sakuma, 1995).

In freely moving EB-treated ovariectomized females, neurons associated with bouts of sexual interactions with a male partner in rate-meter and ethograms have been shown to have a mean firing rate of 10.3 Hz (Kato and Sakuma, 2000). Electrical stimulation of the POA at around 10 Hz suppressed lordosis, with a slow onset and gradual suppression which reached a maximum at 90 min. This effect has also been characterized by slow recovery of lordosis after the termination of POA stimulation (Pfaff and Sakuma, 1979; Takeo et al., 1993). The peculiar time course in the behavioral response to the POA stimulation disappeared by the removal of dorsal connection of the POA by a horizontal knife cut (roof cut), or in particular, the disruption of the stria terminalis, resulting in immediate interruption of lordosis in response to current application (Takeo et al., 1993). Therefore, the POA contains a particular set of neurons that are responsible for the inhibition of lordosis. The elimination of facilitatory neural components for this reflex, which enter the POA via the stria terminalis, is responsible for the prompt and exaggerated stimulation effect in the roof-cut animals (Figure 1).

## The POA and the proceptive behavior

Of great significance in the observed effects of POA lesions on lordosis is that the effects depend on test situation. For instance, Whitney (1986) found that, in a no-exit paradigm when the females were constrained in the vicinity of males, lordosis was enhanced. In exit tests, in which the females could evade male partners, no lordosis was seen as a consequence of the lack of sexual contacts. Thus, the authors concluded that the enhanced lordosis in the lesioned females detected by no-exit tests was not due to any potentiation in the females' preference to engage in sexual interactions with males.

In the rat, sexual interactions are initiated and paced by females in estrus through patterns of approach toward and withdrawal from sexually active males (Erskine, 1989). Emotional state of the females, determined by activity of the medial amygdala, one major source of estrogen-sensitive POA afferents, may regulate this approach and withdrawal (Kondo and Sakuma, 2005). Preoptic implants of estradiol suppress open-field and increase wheel-running activities in ovariectomized female rats (Fahrbach et al., 1985). These behavioral changes have been interpreted to reflect increased anxiety and fear learning together with locomotor activation, the effects, as investigated in knock-out mice (Ogawa et al., 2003), mediated by ERα-positive, but not ERβ-positive, neurons in the POA. In stressed female rats, however, estradiol has been found to decrease anxious behavior on the open field and to enhance radial-arm maze performance (Bowman et al., 2002). Changes in cognitive and emotional activity have been inferred to reflect a general increase in arousal level (Morgan and Pfaff, 2002), with both responses increasing the likelihood of successful reproduction.

## Preoptic locomotor area

Thus, an increased locomotor activity in female rats in estrus embodies enhanced sexual motivation (Quadagno et al., 1972; Swanson and Mogenson, 1981; Mink et al., 1983; Edwards and Einhorn, 1986; Rivas and Mir, 1990; Paredes and Vazquez, 1999), and the POA has been positively identified as a site for estrogen-induced activation of wheel running (Fahrbach et al., 1985) through activation of ERα (Hertrampf et al., 2008). The POA contributes to the rostro-caudal neural axis for the locomotor synergy (Mori et al., 1992) with its projections to the midbrain locomotor region (MLR) (Swanson et al., 1984, 1987). The preoptic locomotor region, from which stepping can be initiated by chemical (Sinnamon, 1987) or electrical (Sinnamon, 1992) stimulation, is situated in the medial portion of the lateral POA (mLPO). The locomotor activity can be consistently reduced by cholinergic activation of the periventricular POA (Brudzynski and Eckersdorf, 1984; Brudzynski and Mogenson, 1986).

In the EB-dependent regulation of locomotor activity, two separate POA projections to the MLR that mediate EB effects have been identified (Takeo and Sakuma, 1995). The female rat POA contains neurons that promote proceptive behavior (Hoshina et al., 1994). Females with lesions of the peripeduncular nucleus, through which fibers with origins in the POA and other subpallidal structures descend to the MLR (Swanson et al., 1984), characteristically failed to show darting and other solicitatory behavior (Pfeifle and Edwards, 1983). An observation that lesions of the accumbens does not modify soliciting activity (Rivas and Mir, 1990) may mean that the POA constitutes an independent entity for solicitatory behavior, because the accumbens activates locomotion through innervation of the POA (Swerdlow et al., 1984). In a male rat engaging in sexual interaction, however, our recent study showed that the shell of the accumbens contains neurons encoding cues or contexts related to sexual behavior, reward-related processing, and the inhibition of sexual behavior after ejaculation (Matsumoto et al., 2012). These results suggest that estrogen inhibits neural impulse flow from the MPO and facilitates that from the lateral POA. The effects of estrogen, when combined, would culminate in increased locomotor activity that is typical of female rats in estrus.

## Projection neurons in the POA

Stereotaxic infusion of ibotenic acid, an excitotoxin which obliterates POA neuronal soma but spares local axons of passage, enhances lordosis by lowering the threshold for EB needed to induce the reflex (Hoshina et al., 1994). At the same time, females with the excitotoxin lesion did not commit themselves to sexual interactions. Far from showing solicitation, these females antagonized and vigorously resisted any males that attempted to mount them in the non-exit test paradigm. Meanwhile, gradual and persistent suppression of the lordosis reflex followed electrical stimulation of the local axons of passage that survived the excitotoxic damage. Apart from the fact that the females with the POA lesion needed less estrogen to obtain comparable prestimulation quotients with the controls, the lesioned and control animals responded similarly to the stimulation.

In the females with ibotenic-acid lesion of the POA, an additional roof cut dorsal to the POA abolished the stimulus-bound suppression of lordosis, and the stimulation effect was thus due to the activation of axons of passage that presumably descend from the septum, cingulate cortex, or other structures. As described above, the septum is an origin of lordosis-inhibiting efferents (Yamanouchi and Arai, 1990). Thus, the POA is a major target for EB in eliciting proceptive behavior; local POA neurons as well as septal efferents appear to inhibit the lordosis, a receptive behavior.

## Descending projection of the ventral tegmental area

The midbrain ventral tegmental area (VTA) is one of major projection targets of estrogen concentrating neurons in the POA (Fahrbach et al., 1986). Earlier anterograde tracing studies in the rat (Conrad and Pfaff, 1976) and gerbil (Finn et al., 1993) visualized dense POA projection to the VTA. POA projection may in turn activate both ascending and descending efferents of the VTA (Simon et al., 1979a,b; Matsumoto et al., 2012). Electrical stimulation of the VTA in EB-primed ovariectomized female rats caused immediate and strong interruption of lordosis reflex in response to either male mounts or manual cutaneous stimuli. The intensity and the time course of the disruption bore a resemblance to that induced by POA stimulation in the rat with the roof cut. Likewise, lordosis performance returned promptly to the pre-stimulation level after the termination of stimulation. Interestingly, electrical stimulation specifically blocked lordosis without disturbing proceptive behavior. Pharmacological depletion of dopamine did not affect the stimulation on lordosis.

The VTA disruption of lordosis is a result of an activation of a pathway inhibitory to the reflex arc at the lower brainstem. Indeed, non-dopaminergic descending projections of the VTA have been traced ipsilaterally to the ventral and dorsal tegmental nucleus and the ventral CG (Simon et al., 1979a).

Functional demarcation exists between the dorsal and ventral parts of the CG. Opposite patterns of cardiovascular changes have been found to be elicited from lateral and ventrolateral subregions of the CG (Bandler and Shipley, 1994; Vaughan et al., 1996). Activation of CG sites lateral to the aqueduct produced increased arterial pressure and tachycardia; activation of sites ventrolateral to the aqueduct produced decreased arterial pressure and bradycardia. The lordosis reflex is under a similar antagonistic regulation: the dorsal CG is a target of VMN projection, from which the reflex can be promoted. The ventral CG contains descending VTA axons-of-passage, which inhibits the reflex, and electrical stimulation of this structure elicits antidromic action potentials in VTA neurons (Sakamoto et al., 1993) (**Figure 3**). One of the targets of the VTA projection, the dorsal tegmentum, contains neurons associated with paradoxical sleep (Torterolo et al., 2002). Paradoxical sleep is characterized by somatic muscle atonia (Sakai and Neuzeret, 2011), which would result in the disruption of lordosis.

Consistent with morphological studies, POA neurons have been found to be antidromically driven from the VTA (Hasegawa and Sakuma, 1993). Whereas EB treatment decreased antidromic activation threshold for VMN neurons by CG stimulation (Sakuma, 1984), EB showed an opposite effect on the threshold for activation of POA neurons from the VTA. Besides, in both projections, the authors found that EB was effective in females or neonatally orchidectomized males but not in females given testosterone as pups. EB-induced excitability changes in either VMN or POA axons were observed in the ovariectomized females and neonatally orchidectomized males, but not in androgenized females, in parallel with the capability of EB treatment to induce lordosis.

Changes in antidromic activation thresholds, along with those in refractory periods and axonal conduction velocity, indicate an altered axonal excitability. Our experiment in a model system deploying GT1-7 cells showed that EB at physiological doses, that is 100–300 pM in the medium, enhanced Ni^2+^-, Cd^2+^-sensitive BK current after 3 days in culture. BK or KCNM channels have a large conductance, and are voltage-gated. Thus, in this model, the enhanced expression of these channels would decrease excitability (Nishimura et al., 2008).

## Different subsets of POA neurons

**Figure 2:**
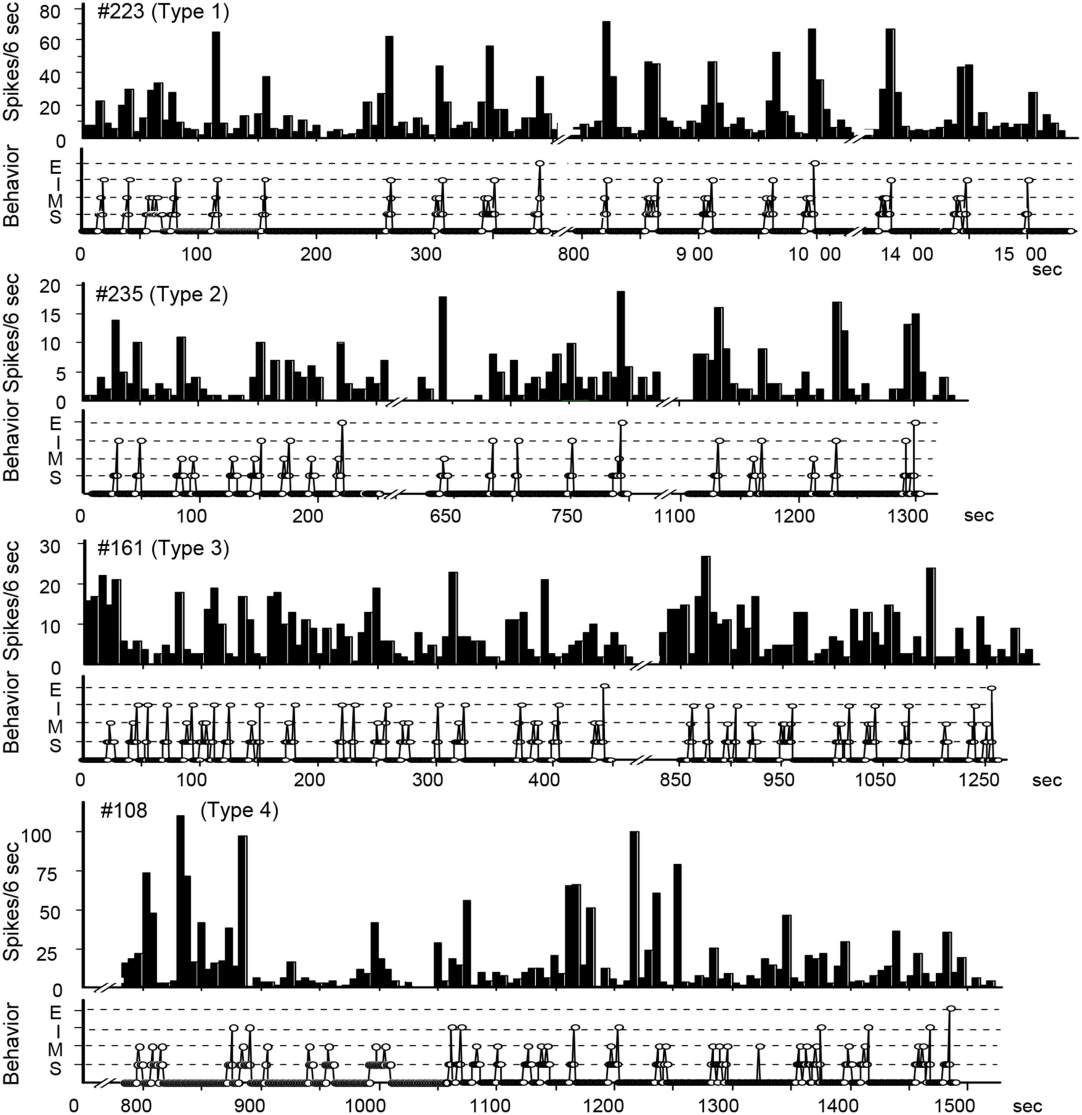
**Temporal relationship between neuronal activity and sexual behavior in rate-meter and ethogram charts**. The activity of these POA neurons was related to bouts of sexual interactions, which were characterized into four types in peri-event histograms associated with different events in sexual behavior (see text).

In order to clarify whether separate POA neurons regulate solicitatory and receptive components of female rat sexual behavior, single unit activities were recorded (Kato and Sakuma, 2000) (Figure 2). Perievent histograms identified separate groups of neurons that increased their firing rate (1) during the solicitatory period, from the initiation of solicitatory locomotion to the male mounts, (2) when the male mounted, or (3) in response to intromission. There was also another set of neurons that were quiescent prior to and throughout the display of the lordosis. Neurons associated with proceptive behavior and somatosensory responses were recorded from the transitional region between the medial and lateral POAs. Those neurons that behaved exactly as if they inhibited the execution of the lordosis were located medially in the medial POA to other neurons. These results thus showed separate sets of POA neurons each specifically associated with proceptive and receptive components of female rat sexual behavior.

## VTA neurons are excited by POA efferents

**Figure 3:**
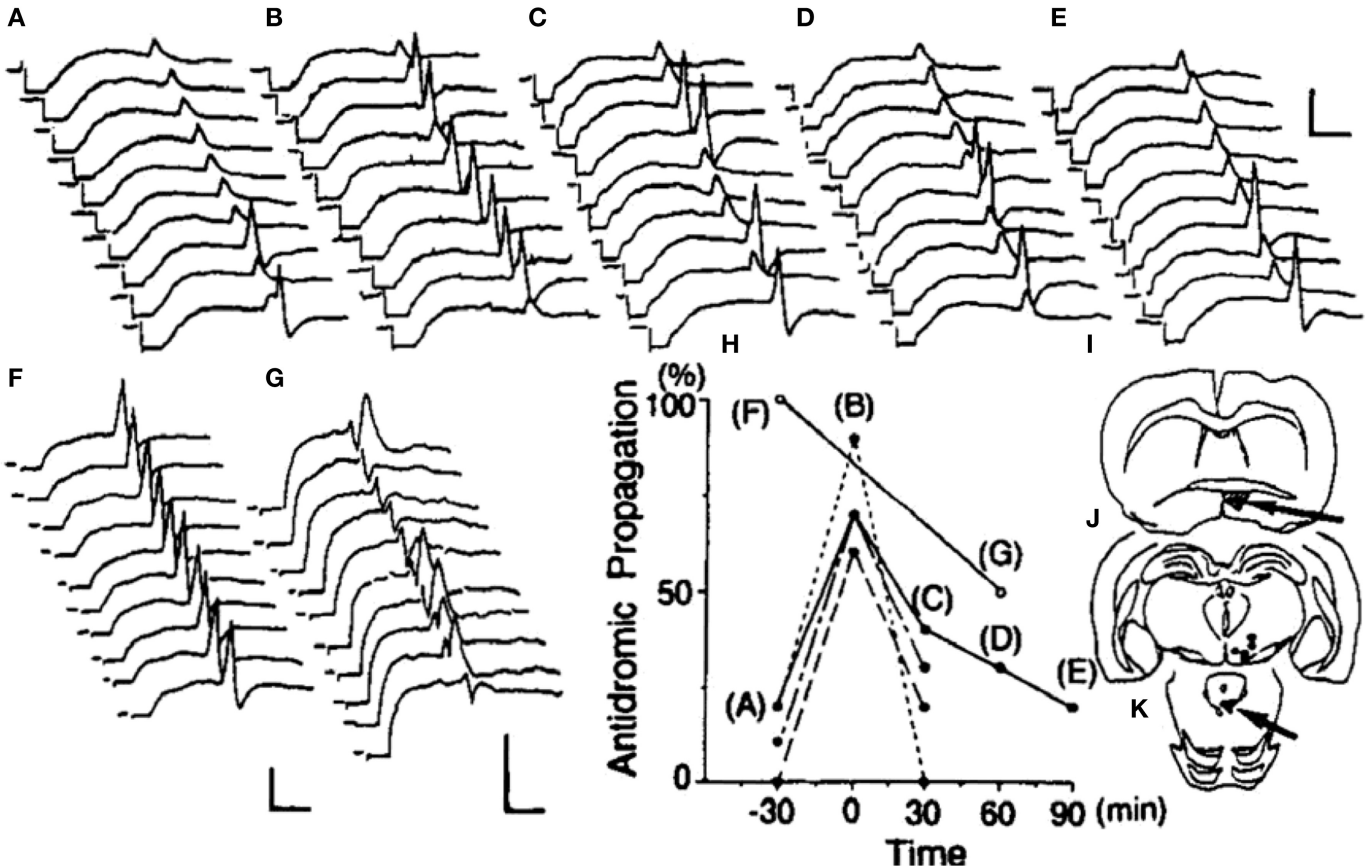
**Effects of POA stimulation or lesion on the propagation of CG-induced antidromic potentials into ventral tegmental area neurons**. Trains of antidromic stimuli were given at 0.5 Hz, and excerpts of the responses in an ovariectomized, estrogen-treated rat are shown **(A–E)**. The POA was stimulated at 100 Hz for a 30-s period during the period indicated in **(B)**. Acute electrolytic lesion made during the period indicated in **(D)** had no effect on this neuron. **(F)** and **(G)** depict recordings from an ovariectomized, non-treated animal that were made before and after an electrolytic lesion of the POA, respectively. As summarized in **(H)**, all four cells in ovariectomized, EB-treated animals originally showed low rates of antidromic propagation that were temporarily increased by POA stimulation. A POA lesion in an ovariectomized female rat, which originally showed a high rate of antidromic propagation, exerted a contrasting effect to that of POA stimulation, resulting in a decrease in the frequency and a delay in antidromic propagation. The position of tip of each stimulation electrode and the extent of the POA lesion are shown in **(I)**, the location of each recorded neuron in **(J)**, and the antidromic stimulation sites in **(K)**. Calibrations are 5 ms and 1 mV (Sakamoto et al., 1993).

In urethane-anesthetized, EB-treated ovariectomized rats, antidromic action potentials elicited in VTA neurons by CG stimulation often terminated at the initial segment and rarely invaded the neuronal soma (Sakamoto et al., 1993) (Figures 3A–K). The authors also found that POA stimulation increased the probability of successful antidromic invasion up to 90%. Conversely, ovariectomized females showed almost 100% success of antidromic invasion without POA stimulation in the absence of EB; acute electrolytic destruction of the POA decreased the invasion rate down to 50%. Thus, the POA is thought to excite the soma of VTA neurons, and EB decreases the impact of POA effect on the VTA. EB would thus decrease the efficacy of neural transmission from the POA to the CG. The pattern of estrogen-induced changes in the excitability of these descending VTA neurons is that required for the behavioral disinhibition of the lordosis reflex.

## Projections to the medulla

The gigantocellular nucleus of the medullary reticular formation (NGc) and lateral vestibular nucleus (LVN) are the origins of the ipsilateral reticulospinal and vestibulospinal tract, respectively, which innervate spinal motoneurons responsible for the induction of the lordosis. Lesion studies have suggested that the contribution of these tracts is not dependent upon the integrity of the other, and that the magnitude of the lordosis deficit is instead correlated with amount of giant cell loss in NGc and Deiters cell loss in the LVN (Modianos and Pfaff, 1979). Finally, lordosis is facilitated by electrical stimulation of the LVN (Modianos and Pfaff, 1977).

Electrical stimulation of the NGc in urethane-anesthetized female rats induced antidromic activation in neurons in the CG. Antidromically driven cells were in all parts of the CG and adjacent mesencephalic reticular field except within the inner ring of the CG that surrounds the aqueduct.

As with the antidromic potentials induced in the VTA in response to CG stimulation, POA stimulation reduced the rate of successful propagation of NGc-induced antidromic potentials into the soma, whereas VMN stimulation increased the rate. Thus, the pattern of descending effects originating in the EB-sensitive POA and VMN on these CG neurons is required for their control of the lordosis reflex, via the regulation of the activity of medullospinal neuron that govern the contraction of back muscles responsible for the induction of the lordosis reflex.

### Conflict of interest statement

The author declares that the research was conducted in the absence of any commercial or financial relationships that could be construed as a potential conflict of interest.
